# High-Performance Liquid Chromatography–Hydrogen/Deuterium Exchange–High-Resolution Mass Spectrometry Partial Identification of a Series of Tetra- and Pentameric Cyclic Procyanidins and Prodelphinidins in Wine Extracts

**DOI:** 10.1021/acs.jafc.9b06195

**Published:** 2020-01-13

**Authors:** Vakarė Merkytė, Edoardo Longo, Michaël Jourdes, Alicia Jouin, Pierre-Louis Teissedre, Emanuele Boselli

**Affiliations:** † Faculty of Science and Technology, Free University of Bozen-Bolzano, Piazza Università 5, 39100 Bozen-Bolzano, Italy; ‡ Oenolab, NOI Techpark South Tyrol, Via Alessandro Volta 13B, 39100 Bolzano, Italy; § Unité de Recherche Œnologie, EA 4577, USC 1366 INRA, ISVV, Université de Bordeaux, 33882 Villenave d’Ornon, France

**Keywords:** cyclic proanthocyanidins, crown proanthocyanidins, red wine, isotopic exchange mass spectrometry, solid-phase extraction

## Abstract

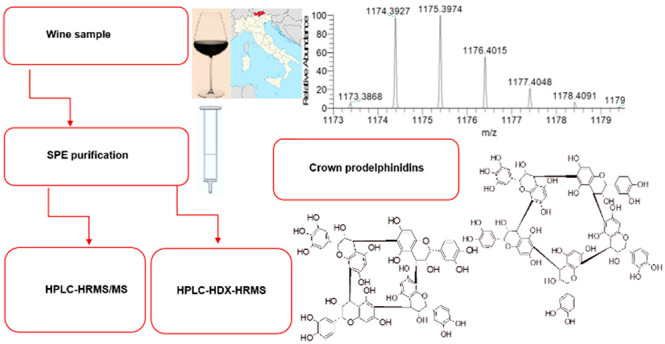

A solid-phase extraction method was applied for the identification of a series of unconventional crown (macrocyclic) B-type proanthocyanidin tetramers (*m*/*z* 1169.2557, 1185.2507, 1201.2456, and 1217.2405) and pentamers (*m*/*z* 1457.3191, 1473.3140, 1489.3090, 1505.3039, and 1521.2988) containing (epi)catechins only (procyanidins) or (epi)catechins and (epi)gallocatechins (prodelphinidins). These compounds were identified in red wine by high-performance liquid chromatography–high-resolution mass spectrometry coupled with online hydrogen/deuterium exchange (HDX) after purification with a C18 solid-phase extraction phase from the original wine sample. The number and type of monomer units present in each procyanidin and prodelphinidin are discussed on the basis of the experimental measured masses, their retention time distribution among observed isomers, tandem mass spectrometry fragmentations, and the HDX-induced shift of the theoretical monoisotopic mass. The elution in reverse-phase high-performance liquid chromatography shifted to lower retention times when the ratio of (epi)gallocatechin units in these molecules increased with respect to the content of (epi)catechin units, as a consequence of the increase of polarity.

## Introduction

An unusual cyclic B-type procyanidin (PC) tetramer was recently identified in wine, showing an uncommon macrocyclic molecular structure. Its structure was assigned by nuclear magnetic resonance (NMR) and confirmed by mass spectrometric approaches.^1−3^ In particular, online hydrogen/deuterium exchange (HDX) coupled to high-resolution mass spectrometry (HRMS) allowed for the identification of one tetrameric, one pentameric, and one hexameric B-type macrocyclic PC in wine (crown procyanidins). The method allowed for the unambiguous differentiation of the cyclic B-type PC from their non-cyclic (conventional) A-type PC analogues, which are in effect regioisomers (considering one A linkage) and, therefore, indistinguishable by HRMS alone.^1^ In addition, all of these cyclic congeners were shown to possess higher polarities and a lower degree of fragmentation upon tandem mass spectrometry (MS/MS) experiments than their (conventional) non-cyclic B- and A-type analogues.^2,3^ Notably, the cyclic tetramer PC was also identified in a cranberry extract, where only its isomeric A-type PC analogue was expected, unlike what was reported in previous literature.^1^ Although it is not as detailed as NMR for structural resolution, HDX applied to high-performance liquid chromatography (HPLC)–HRMS is much less time-consuming and showed the ability of exploring other possible cyclic proanthocyanidin (PAC) congeners. In fact, two cyclic prodelphinidins (PDs) were identified in wine.^4^ Indeed, the very diverse flavan-3-ol monomeric class produces a high number of possible different theoretical proanthocyanidins (PACs) classifiable by the (1) number of monomers, (2) types of condensed monomers, (3) stereo- and regioisomers (with usually two stereogenic carbons per monomer, C2 and C3, besides the chiral C4, which itself assumes either α or β configurations when linked to the next monomer unit), (4) theoretically equally possible C4–C6 or C4–C8 linkages, (5) overall cyclic (crown) or non-cyclic macrostructure, and (6) presence of one or more A-type linkages. Although not all combinations are known in nature, the complexity of structures in tannin-rich red wine is particularly high.^5,6^

The aim of the present work is to complete the identification of the most abundant cyclic prodelphinidins in wine,^4^ which has not been feasible before for most of them because of their low concentrations. Thus far, these compounds have been observed applying different analytical approaches [with or without a preliminary isolation, for example, after solid-phase extraction (SPE)], with the only limitation being their concentration in wine if no extraction was applied.^1,2^ In fact, white wines required a 10:1 reconcentration for detecting the cyclic oligomers;^11^ instead, in red wines, the most concentrated of these compounds (e.g., the cyclic tetrameric procyanidin) was observed with no special effort, reconcentration, extraction, or concentration after SPE.^1^ In addition, the preparative isolation of the most abundant of the cyclic tetrameric procyanidin and its full structural resolution have already been achieved.^2^ Recent reports also showed a relationship between the proportion of cyclic congeners with the grape variety, which is appealing toward the investigation of novel wine quality markers,^11^ suggesting, in turn, that the formation of these compounds was directly in the grape. SPE has been applied to red wine as a sample preparation approach to obtain preconcentrated and cleaner samples containing the cyclic oligomers. Eventually, the identification of a higher number and variety of congeners may represent an effective tool for wine quality and authenticity assessment. This was already partially observed in wines obtained from specific grape varieties.^4,11^

## Experimental Section

### Material

Liquid chromatography–mass spectrometry (LC–MS)-grade solvents (acetonitrile), deuterium oxide 99.9%_D_, and additives (formic acid, formic acid-*d*_2_, ammonium acetate, and ammonium acetate-*d*_7_) were obtained from Sigma-Aldrich Srl (Milano, Italy). Milli-Q water was produced in-house (18.2 MΩ, Sartorius Arium mini, Sartorius Italy Srl, Varedo, MB, Italy). A sample of Lagrein wine (2016 harvest) used to develop the SPE method was supplied by a local winery (Kellerei Bozen, Bolzano, Italy).

### SPE Purification of Wine Proanthocyanidins

SPE purification of red wine was performed following an adapted published method.^7^ Briefly, the samples were concentrated under vacuum and recovered with milli-Q water (10 times the initial concentration).^1,4,11^ Then, three 1 g SPE C18 cartridges (6 mL each, ASPEC C18, 40–63 μm, lot number 66597, Gilson, Inc., Middleton, WI, U.S.A.) were conditioned with methanol (2 mL), followed by milli-Q water (2 mL). The concentrated wine aliquots (2 mL/each) were gently poured in the cartridges. After the wine was well-absorbed onto the top layer of the resin, all residual liquid was dried with a gentle N_2_ flux. Each cartridge was then gently (not to disrupt the top layer of the sorbent material loaded with the sample) washed with 5%_aq_ formic acid in acetonitrile (10 mL, fraction F1). The cartridges were subsequently washed with 0.1% formic acid in methanol (fraction F2). After the addition of pure formic acid (300 μL/cartridge), each cartridge was washed with 95% methanol (10 mL, fraction F3). All fractions were eventually concentrated at reduced pressure (9 mbar) at 30 °C, then recovered, and redried several times with gradient phase B (deuterated or not) to remove water/ethanol traces, followed by a further gentle drying with N_2_ to dryness. The complete solvent removal was monitored by weighing. Dried F1, F2, and F3 weighed 93.6, 20.1, and 18.0 mg, respectively. The sample to be analyzed (the entire dry F3) was redissolved in the pure chromatographic phase A (0.1%, v/v, formic acid in 0.02 mol L^–1^ ammonium acetate in water, 250 μL, with a final concentration of F3 for the injection of 72 mg mL^–1^) and directly used for the injection. When HDX was performed, the samples were eventually reconstituted in 5% deuterated phase B in deuterated phase A (0.1%, v/v, deuterated formic acid in 0.02 mol L^–1^ fully deuterated ammonium acetate in deuterium oxide, 250 μL) and the rewashing/recovering steps were performed several times only with deuterated solvents to achieve a complete HDX of all OH protons. The sample to be analyzed (the entire dry F3) was redissolved in the pure deuterated chromatographic phase A (250 μL) and directly used for the injection.

### HPLC–HRMS/MS Analysis

The HPLC–HRMS/MS method was adapted from published reports.^1,8−16^ The apparatus consisted of a Q Exactive HRMS mass spectrometer (Thermo Fisher Scientific, Rodano, Milano, Italy) coupled with Agilent 1260 HPLC (Agilent Technologies Italia S.p.A., Cernusco sul Naviglio, Milano, Italy). The elution was performed at a flow rate of 1 mL min^–1^ with a C18 LC column (ODS Hypersyl, 125 × 4.6 mm inner diameter, 5 μm, Thermo Scientific) protected with a guard column filter (ODS Hypersil, 5 μm pore size, 10 × 4 mm drop-in guards, Thermo Fisher Scientific). The mobile phases consisted of solvent A (0.1%, v/v, formic acid in 0.02 mol L^–1^ ammonium acetate in water or 0.1%, v/v, deuterated formic acid in 0.02 mol L^–1^ fully deuterated ammonium acetate in deuterium oxide) and solvent B (0.1%, v/v, formic acid in saturated ammonium acetate in acetonitrile or 0.1%, v/v, deuterated formic acid in saturated fully deuterated ammonium acetate acetonitrile, LC–MS grade). The gradient timetable was from 5 to 25% B (v/v) from 0 to 21 min, then from 25 to 95% B (v/v) from 21 to 22 min, then 95% B until 27 min, and then from 95 to 5% B (v/v) from 27 to 28 min, followed by 5% B (v/v) until 32 min.

### Full MS Parameters

Heated electrospray ionization in positive ion mode (H-ESI+) parameters were as follows: sheath gas at 20 (arbitrary units), auxiliary gas at 5 (arbitrary units), auxiliary temperature at 250 °C, spray voltage at +3.8 kV (+4 kV with deuterium oxide), capillary temperature at 320 °C, and radio frequency (RF) S-lens at 70. Mass range = *m*/*z* 500–2000 with a set resolution of 70 000 [at *m*/*z* 200 automatic gain control (AGC) target at 3 × 10^6^ and maximum injection time of 300 ms]. Data-dependent (dd)-LC–MS/MS experiments were run on the N_2_-concentrated samples, with full MS parameters unchanged, MS/MS AGC target at 10^6^, maximum injection time at 300, FT-MS set resolution at 35 000, loop count at 5, isolation window at *m*/*z* 2 or 3 with *m*/*z* 1 offset, and normalized collision energy at 15%. For data-dependent settings, the minimum AGC target was 3 × 10^3^, apex trigger was 2–8 s, charge exclusion was 3–8 and higher, and “if idle” tool was set to “do not pick others”. Lock masses were constantly employed. When deuterium oxide was employed, the lock masses were modified accordingly. The MS data and results were collected and analyzed with Xcalibur 3.1 software and Compound Discoverer 2.0 (Thermo Scientific). XLStat (version 2016.02.28430, Addinsoft, Paris, France) and The Unscrambler (version 10.4.43636.111, CAMO Software AS., Oslo, Norway) software were employed for the statistical analysis. Relative abundances plot versus number of (epi)gallocatechins and fittings were obtained by Origin 2016 software (OriginLab Corporation, Northampton, MA, U.S.A.).

## Results and Discussion

### SPE Purification of the Wine PAC Fraction

The applied SPE method was optimized from the literature to provide a concentrated mixture on the proanthocyanidin fraction only, devoid of the other wine components.^7^ Our aim was to overcome the limitations observed in the identification and HPLC–MS characterization of these compounds in wine,^1,4,11^ namely, the low relative abundance observed even with a concentration factor of 10:1 from the native sample. In non-purified wine samples, the amount of these compounds was roughly estimated to be about ^1^/_10_ of their non-cyclic congeners.^11^ For the profile of PAC in the 10:1 concentrated wine (also from several grape varieties) and analyzed with the same HPLC–HRMS method, we refer to previous reports.^1,4,11,13^ Previously, only three cyclic procyanidins and two cyclic prodelphinidins (the most abundant compounds) could be observed. The three SPE fractions were monitored by HPLC–HRMS (same method applied for PAC analysis). PACs were identified exclusively in the F3 fraction (small traces for some of the most abundant compounds eluting in F3 were observed also in F2, probably as a result of overloading). The fractions F1 and F2 contained simple phenolics and other non-phenolic fractions; therefore, they will not be discussed further.

### HPLC–HRMS Analysis of the F3 Fraction

**Figure 1:**
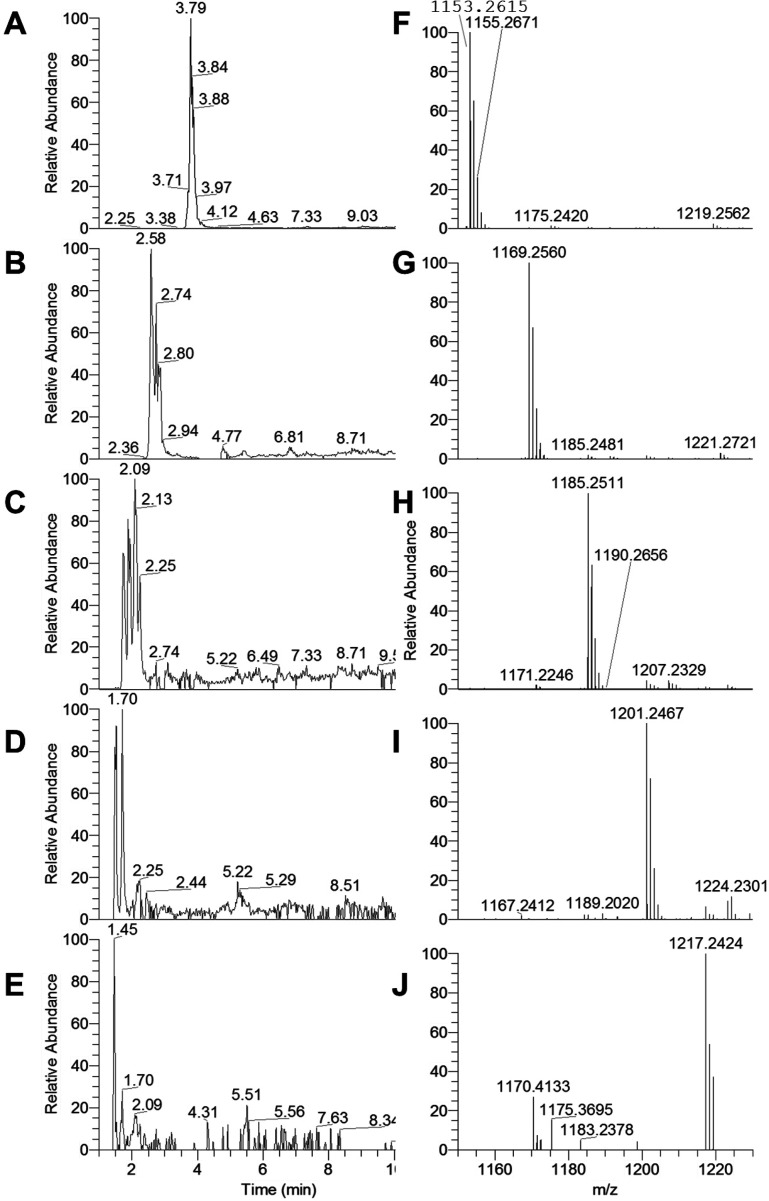
Extracted ion chromatograms for tetrameric (A) *m*/*z* 1153.2608 {0 (epi)gallocatechins, [C_60_H_48_O_24_ + H]^+^}, (B) *m*/*z* 1169.2557 {1 (epi)gallocatechin, [C_60_H_48_O_25_ + H]^+^}, (C) *m*/*z* 1185.2507 {2 (epi)gallocatechins, [C_60_H_48_O_26_ + H]^+^}, (D) *m*/*z* 1201.2456 {3 (epi)gallocatechins, [C_60_H_48_O_27_ + H]^+^}, and (E) *m*/*z* 1217.2405 {4 (epi)gallocatechins, [C_60_H_48_O_28_ + H]^+^}. Full MS (ESI+ , 6 amu range shown) for tetramers at (F) 3.8 min (for panel A), (G) 2.6 min (for panel B), (H) 1.9 min (for panel C), (I) 1.7 min (for panel D), and (J) 1.4 min (for panel E). A 4 ppm filter was applied to the extracted ion chromatograms.

**Figure 2:**
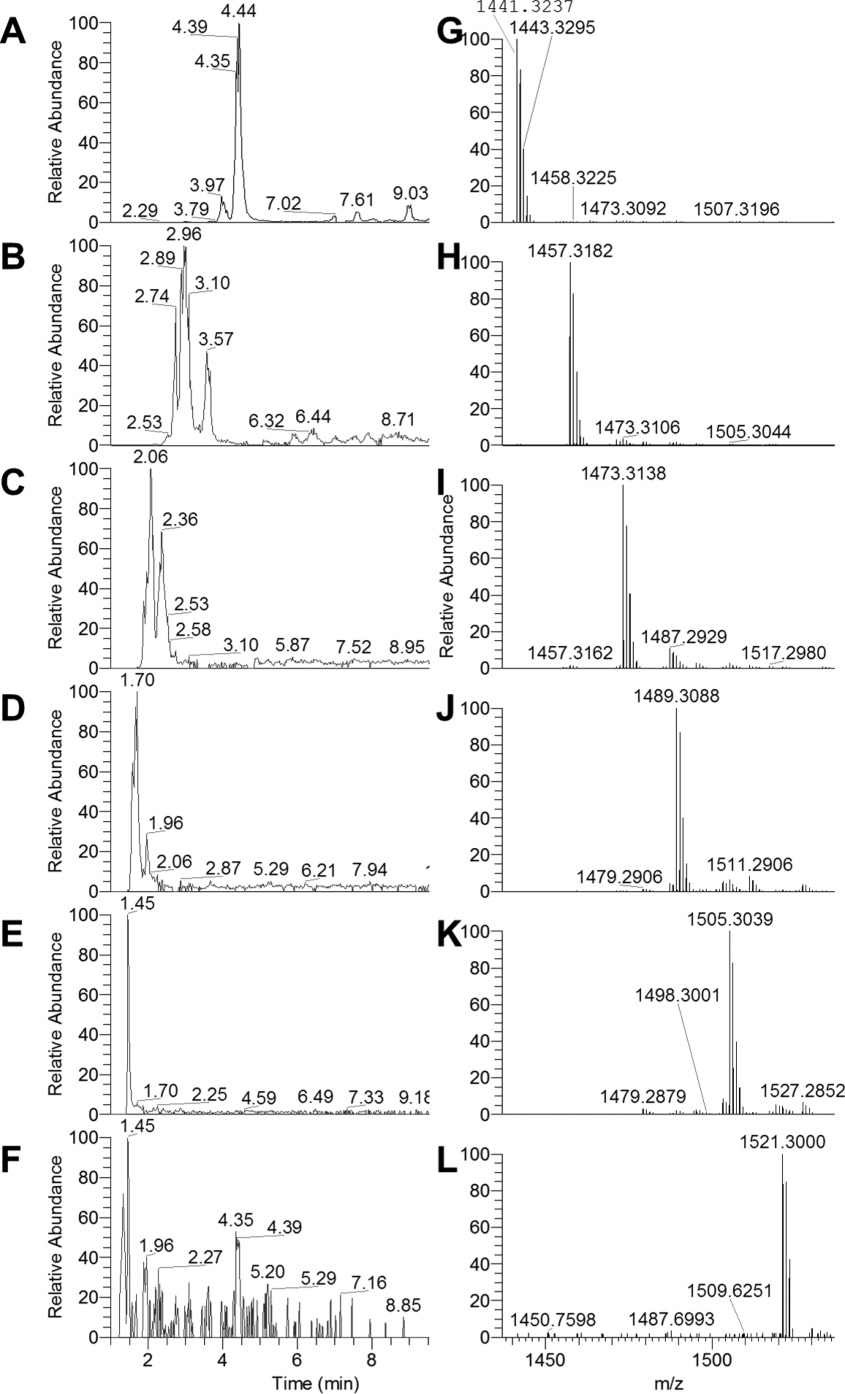
Extracted ion chromatograms for pentameric (A) *m*/*z* 1441.3242 {0 (epi)gallocatechins, [C_75_H_60_O_30_ + H]^+^}, (B) *m*/*z* 1457.3191 {1 (epi)gallocatechin, [C_75_H_60_O_31_ + H]^+^}, (C) *m*/*z* 1473.3140 {2 (epi)gallocatechins, [C_75_H_60_O_32_ + H]^+^}, (D) *m*/*z* 1489.3090 {3 (epi)gallocatechins, [C_75_H_60_O_33_ + H]^+^}, (E) *m*/*z* 1505.3039 {4 (epi)gallocatechins, [C_75_H_60_O_34_ + H]^+^}, and (F) *m*/*z* 1521.2988 {5 (epi)gallocatechins, [C_75_H_60_O_35_ + H]^+^}. Full MS (ESI+, 6 amu range shown) for pentamers at (G) 4.4 min (for panel A), (H) 3.0 min (for panel B), (I) 2.4 min (for panel C), (J) 1.7 min (for panel D), (K) 1.5 min (for panel E), and (L) 1.3 min (for panel F). A 4 ppm filter was applied to the extracted ion chromatograms.

All of the material collected in fraction F3 has been used for the analysis, to obtain the highest possible concentration and signal for the target analytes; indeed, more extract could be obtained by the same SPE procedure on other wine sample aliquots. The analyses were performed after recovering F3 in the appropriate solvent following the method described in detail in the Experimental Section. The sample was made by dissolving F3 in the pure (non-deuterated or deuterated) solvent phase A and injected as such. The HPLC traces and related extracted ion chromatograms (from the full MS analysis) for the expected theoretical masses of tetra- and pentameric cyclic B-type proanthocyanidins with various flavan-3-ol units are shown in Figures 1 and 2, respectively.

In the presented fFigures (panels A–E of Figure 1 and panel A–F of Figure 2), the extracted ion chromatograms corresponding to the whole series from the cyclic tetra- and pentameric PCs [no (epi)gallocatechins, Figures 1A and 2A, respectively] to the cyclic PD with no (epi)catechin but only (epi)gallocatechins (Figures 1E and 2F, respectively) are reported on the left. The MS spectra corresponding to the shown EIC peaks are presented on their right (e.g., the chromatogram in Figure 1A corresponds to the MS spectrum in Figure 1F, etc.). The same approach has been used for the corresponding deuterated counterparts further on. All cyclic tetra- and pentameric species (c-PC and c-PD) were observed in the red wine extract. Interestingly, no crown hexameric PD was observed. In addition, a minor component appeared in Figure 2F (retention time of 4.35 min) as a possible isobaric compound to the cyclic pentamer PD [5 (epi)gallocatechins] eluting at 1.45 min; however, the signal for that minor species was too small to allow for any further characterization. A discussion about the cyclic tetra-, penta-, and hexameric procyanidins (PCs) was already presented in a previous study^1,11^ as well as for two cyclic PDs [one tetramer and one pentamer with only one (epi)gallocatechin] that were also previously observed for a sample not prepared with SPE (but with a much lower relative abundance as expected).^4^

In this report, 11 cyclic PAC candidates were observed. All identified species were listed also in Table 1 for clarity, along with their measured experimental masses and retention times, in both H_2_O and D_2_O.

**Table 1:** Observed Cyclic PAC Species

species^a^	elemental composition [neutral (H)]	theoretical mass [*m*/*z* (H)]	measured mass [*m*/*z* (H)]	elemental composition [neutral (D)]	theoretical mass [*m*/*z* (D)]	measured mass [*m*/*z* (D)]	retention time [min (H)]	retention time [min (D)]
c-tetramer	C_60_H_48_O_24_	1153.2608	1153.2615	C_60_H_28_D_20_O_24_	1174.3926	1174.3927	3.8	4.7
c-tetramer-1-galloc	C_60_H_48_O_25_	1169.2557	1169.2560	C_60_H_27_D_21_O_25_	1191.3938	1191.3944	2.6, 2.7	3.3, 3.6
c-tetramer-2-galloc	C_60_H_48_O_26_	1185.2507	1185.2511	C_60_H_26_D_22_O_26_	1208.3950	1208.3979	1.7, 1.9, 2.1, 2.3	2.1, 2.4, 2.7, 2.9
c-tetramer-3-galloc	C_60_H_48_O_27_	1201.2456	1201.2467	C_60_H_25_D_23_O_27_	1225.3962	1225.3966	1.6, 1.7	1.7, 2.0
c-tetramer-4-galloc	C_60_H_48_O_28_	1217.2405	1217.2424	C_60_H_24_D_24_O_28_	1242.3974	1242.3983	1.5	1.5
c-pentamer	C_75_H_60_O_30_	1441.3242	1441.3237	C_75_H_35_D_25_O_30_	1467.4874	1467.4865	4.0, 4.4	5.4
c-pentamer-1-galloc	C_75_H_60_O_31_	1457.3191	1457.3182	C_75_H_34_D_26_O_31_	1484.4886	1484.4883	2.7, 2.9, 3.0, 3.6	3.3, 3.8, 3.9, 4.6
c-pentamer-2-galloc	C_75_H_60_O_32_	1473.3140	1473.3138	C_75_H_33_D_27_O_32_	1501.4898	1501.4894	2.1, 2.4^b^	2.5, 2.7, 2.8, 3.1, 3.2
c-pentamer-3-galloc	C_75_H_60_O_33_	1489.3090	1489.3088	C_75_H_32_D_28_O_33_	1518.4910	1518.4909	1.7	2.0, 2.5
c-pentamer-4-galloc	C_75_H_60_O_34_	1505.3039	1505.3039	C_75_H_31_D_29_O_34_	1535.4922	1535.4913	1.5	1.6
c-pentamer-5-galloc	C_75_H_60_O_35_	1521.2988	1521.3000	C_75_H_30_D_30_O_35_	1552.4934	1552.4932	1.5	1.4

a The abbreviations used are as follows: l, non-cyclic B-type oligomer; c, cyclic B-type oligomer; and *n*-galloc, number of (epi)gallocatechins in the oligomer.

b Broad overlap (retention times not completely identified).

The observed tetramers contained from 1 to 4 (epi)gallocatechins, and the pentamers contained from 1 to 5 (epi)gallocatechins. Some compounds eluted very early as if these were not in reality retained by the stationary phase [cyclic prodelphinidins with 4 and 5 (epi)gallocatechin monomers]. However, their detection time still reflected quite clearly the elution order imposed by the increasing polarity, which is associated with the increasing proportion of the trihydroxy-substituted flavan-3-ol monomer units [(epi)gallocatechin]. Such a detection order is respected for all compounds presented here. However, any compound anticipating the injection peak itself could not truly be associated with an actual retention time, because these polar species were in fact not properly retained by the stationary phase; however, they were in any case detected by HRMS.

**Figure 3:**
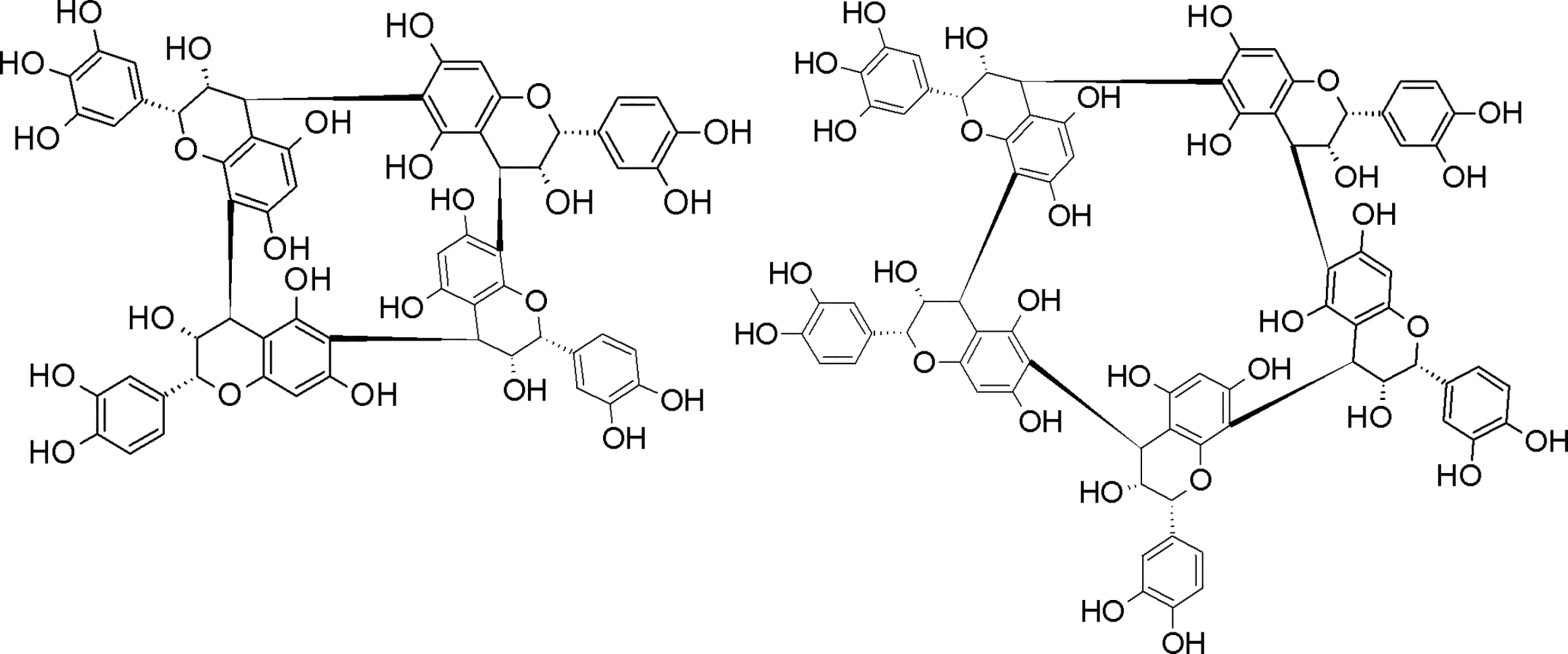
Examples of the investigated compounds: (left) cyclic tetramer PD [1 (epi)gallocatechin] and (right) cyclic pentamer PD [1 (epi)gallocatechin]. In the figure, all of the configurations of the stereogenic centers and all preferences for the intermonomeric B-type linkages reflect the actual structure of the tetrameric procyanidin reported by Zeng et al.,^3^ following similar considerations presented before.^11^ These structural details have not been resolved for the shown compounds.

Different from a previous report where only two of them were identified in several white and red wines,^4^ here, the whole series of prodelphinidins was observed, thanks to the employed SPE purification procedure. A consistent shift toward lower retention times was observed for these species with an increasing proportion of (epi)gallocatechin monomeric units building the “crown” structure. A schematic example of two structures representing a cyclic tetrameric PD and a cyclic pentameric PD, both containing one (epi)gallocatechin, is given in Figure 3.

The linkage preferences shown in Figure 3 (C4–C6 or C4–C8) are only presumed on the basis of the NMR data of the tetrameric crown procyanidin.^3^ However, the arrangement of stereogenic centers was not evaluated for these shown compounds and will require further investigations.

Some crown species displayed high polarities (scarcely retained or even not retained), such as the 4- and 5-mers with more than 3 (epi)gallocatechins (Figures 1 and 2 for tetra- and pentamers, respectively; all of their retention times are grouped between 1.0 and 4.5 min).

The extracted ion chromatograms and MS/MS fragmentations for some of the related non-cyclic analogues are shown in Figures S1–S5 of the Supporting Information. For the non-cyclic PD analogues, identical considerations to those presented for the PC can be made.^1,13^ Namely, in the HPLC–HRMS profiles, the non-cyclic species displayed a number of isobaric peaks always greater than their corresponding cyclic analogues; such a difference is also found for non-cyclic A-type PAC, as shown earlier.^13^ However, different from what was seen with cyclic PC,^1^ with most of the cyclic PD, more than one isomeric peak of comparable relative abundance was observed, in particular, for an intermediate number of (epi)gallocatechins (panels B–D of Figures 1 and 2). It could be argued that the variables most relevant at affecting the cyclic PAC conformation [e.g., the configuration at C3, e.g., with the (+)-catechin versus (−)-epicatechin monomer unit, the C4–C6 or C4–C8 linkage preferences, and the possible C4 α or β configurations] may be likely more limited than the combinations theoretically available. This possibility was confirmed for the highly symmetrical cyclic tetrameric procyanidin by the structural resolution.^11^ On the contrary, the presence of one or more additional OH units on any B ring (a B ring in any monomeric unit constituent) should not be so effective at influencing the overall macromolecular conformation (even if it increases the overall compound polarity) or the probability of undergoing the cyclization mechanism from the (still unknown) precursors. Instead, the presence of very near isobaric (isomeric) species (much closer in retention times than the reported non-cyclic diastereoisomers) for these mixed composition cyclic PD might be reasonably consistent with the possible presence of sequence/positional isomers [the OH groups distinguishing (E)C from (E)GC being present on any possible monomeric unit, which is the same as saying that these isomers have the same proportion of (epi)gallocatechins versus (epi)catechins but a different sequential order], instead of diastereoisomers [also, for the non-cyclic congeners, diastereoisomers show much more spread-out isomeric patterns in their extracted ion chromatogram (EIC) traces using reversed-phase liquid chromatography (RP-LC) separations]. However, apart from these speculative arguments, limited information was achievable to such detail by HDX only and compound isolation and structural characterization (e.g., NMR analysis) will be required.

**Figure 4:**
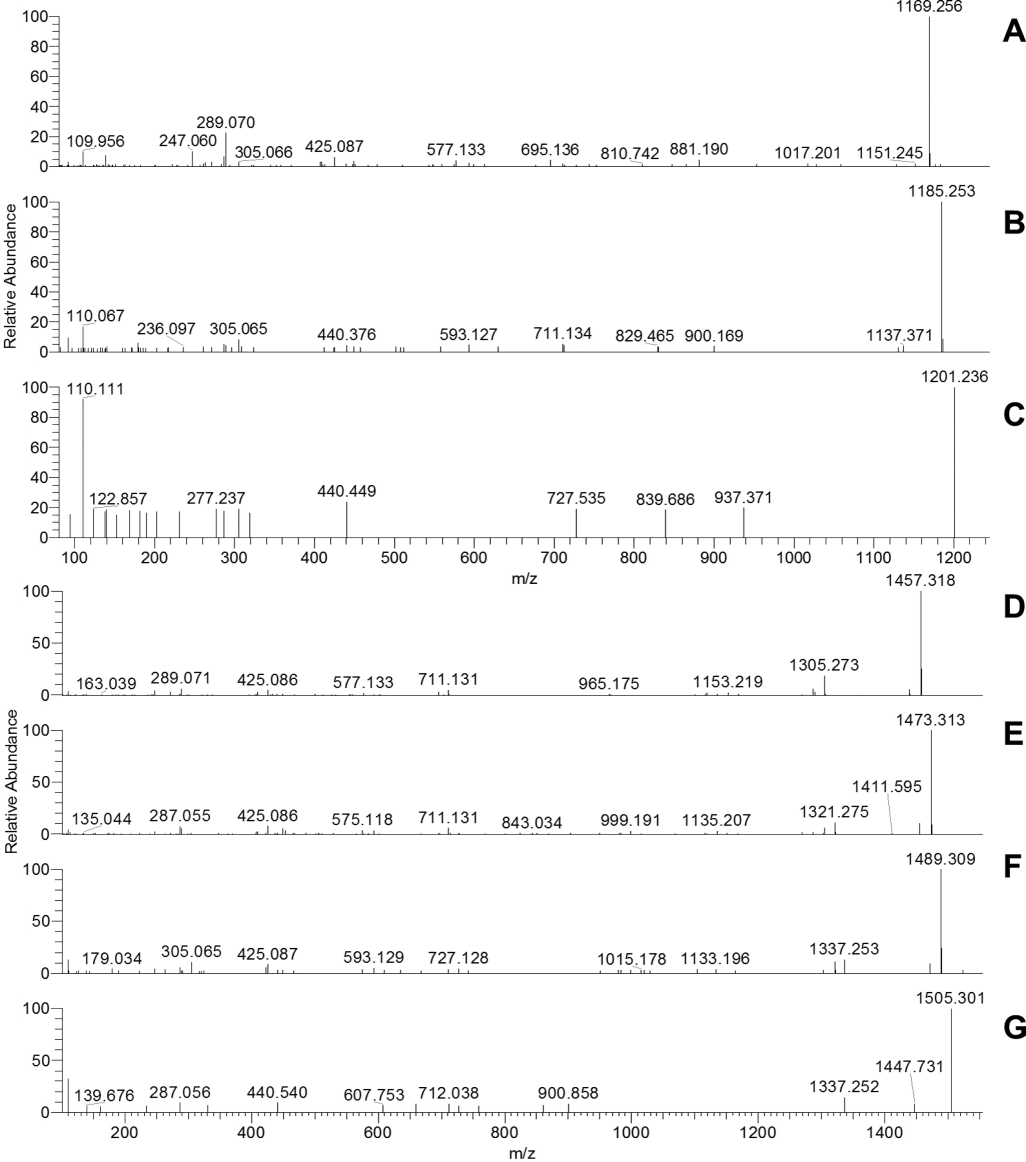
MS/MS spectra of tetrameric (A) *m*/*z* 1169.256 [1 (epi)gallocatechin], (B) *m*/*z* 1185.251 [2 (epi)gallocatechins], and (C) *m*/*z* 1201.246 [3 (epi)gallocatechins] and pentameric (D) *m*/*z* 1457.319 [1 (epi)gallocatechin], (E) *m*/*z* 1473.314 [2 (epi)gallocatechins], (F) *m*/*z* 1489.309 [3 (epi)gallocatechins], and (G) *m*/*z* 1505.304 [4 (epi)gallocatechins]. Spectra were averaged over 0.5 min around the center of the detected peaks.

A further confirmation of the B-type structure for the most abundant PD was obtained by MS/MS fragmentation experiments, which are reported in Figure 4.

As observed earlier with PC, the degree of fragmentation during MS/MS experiments is one of the features most distinguishing these cyclic species from their non-cyclic congeners.^1,13^ The use of low normalized collision energies (NCEs) (herein, 15%) was useful to better see the different behaviors of all of the crown PAC versus their linear PAC congeners.^1^ The crown PAC showed, in general, a much higher resistance to depolymerization, either induced under MS/MS fragmentation conditions or during acidic conditions, as previously reported.^1,3^ In addition, the fragmentation preferences during fragmentation experiments differed between the cyclic and non-cyclic congeners, because the cyclic congeners undergo B-linkage cleavage [quinone methide (QM) mechanism] less preferably than their non-cyclic congeners (as shown also in previous literature for procyanidins),^1^ showing instead a marked preference for retro-Diels–Alder (RDA) fragmentations from the respective molecular ions (for example, for the compounds presented here: *m*/*z* 1017.20 ← 1169.26, *m*/*z* 1305.27 ← 1457.32, *m*/*z* 1321.27 ← 1473.32, and *m*/*z* 1337.25 ← 1489.31).^1,4^ Interestingly, the corresponding RDA fragments from the molecular ions (loss of 152 Da) were instead not at all observed with any of the non-cyclic congeners (Figures S1–S5 of the Supporting Information). Again, this is in line with previous observations,^1^ and it shows that QM is prevalent for non-cyclic PAC, in general, whereas it contributes much less with the macrocyclic compounds. Examples of fragments produced by the QM mechanism are however present, although the intensities are negligible. For example, in Figure 4A [cyclic tetrameric prodelphinidin with 1 (epi)gallocatechin], it is possible to observe a *m*/*z* 881.19 ← 1169.29 {loss of a (epi)catechin moiety + 2[H]} and a *m*/*z* 577.13 ← 881.19 {loss of a (epi)gallocatechin moiety + 2[H]}. The peaks *m*/*z* 305.07 and 289.07 are instead the fragments corresponding to monomers {peaks of (epi)catechin – 2[H] and (epi)gallocatechin – 2[H], respectively}, and these can be observed in all spectra. In Figure 4D, it is possible to observe the fragment *m*/*z* 1153.22 ← 1457.31, corresponding to the loss of the (epi)gallocatechin monomer from the prodelphinidin pentamer with 1 (epi)gallocatechin + 2[H]. An illustration of the QM and RDA fragmentation mechanisms is reported in Figure S6 of the Supporting Information.^17,18^

**Figure 5:**
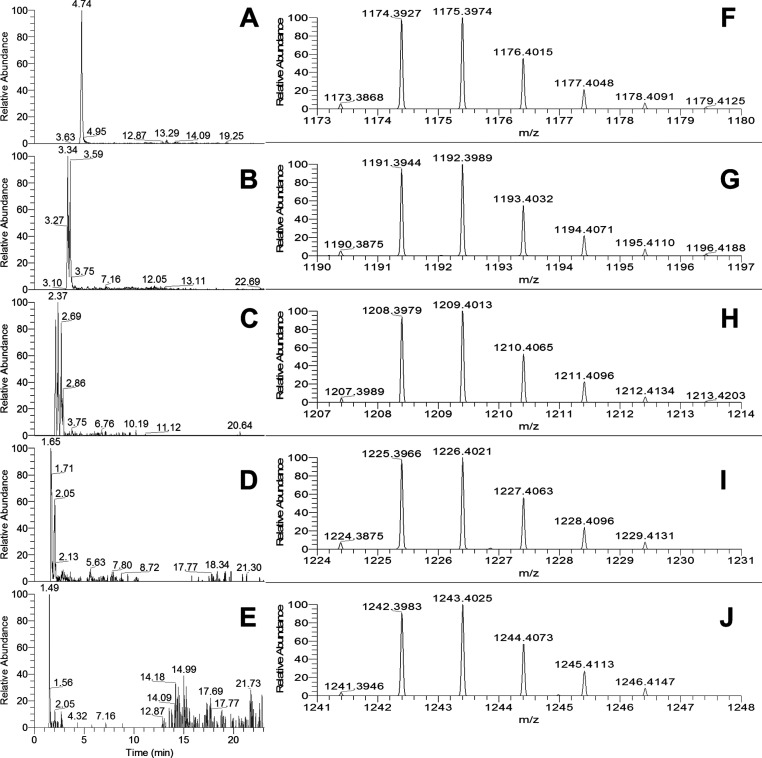
Extracted ion chromatograms in deuterium oxide for tetrameric (A) *m*/*z* (D) 1174.3926 {0 (epi)gallocatechins, [C_60_H_28_D_20_O_24_ + D]^+^}, (B) *m*/*z* (D) 1191.3938 {1 (epi)gallocatechin, [C_60_H_27_D_21_O_25_ + D]^+^}, (C) *m*/*z* (D) 1208.3950 {2 (epi)gallocatechins, [C_60_H_26_D_22_O_26_ + D]^+^}, (D) *m*/*z* (D) 1225.3962 {3 (epi)gallocatechins, [C_60_H_25_D_23_O_27_ + D]^+^}, and (E) *m*/*z* (D) 1242.3974 {4 (epi)gallocatechins, [C_60_H_24_D_24_O_28_ + D]^+^}. Full MS for tetramers at (F) 4.7 min (for panel A), (G) 3.3 min (for panel B), (H) 2.4 min (for panel C), (I) 1.6 min (for panel D), and (J) 1.5 min (for panel E). A 4 ppm filter was applied to the extracted ion chromatograms.

**Figure 6:**
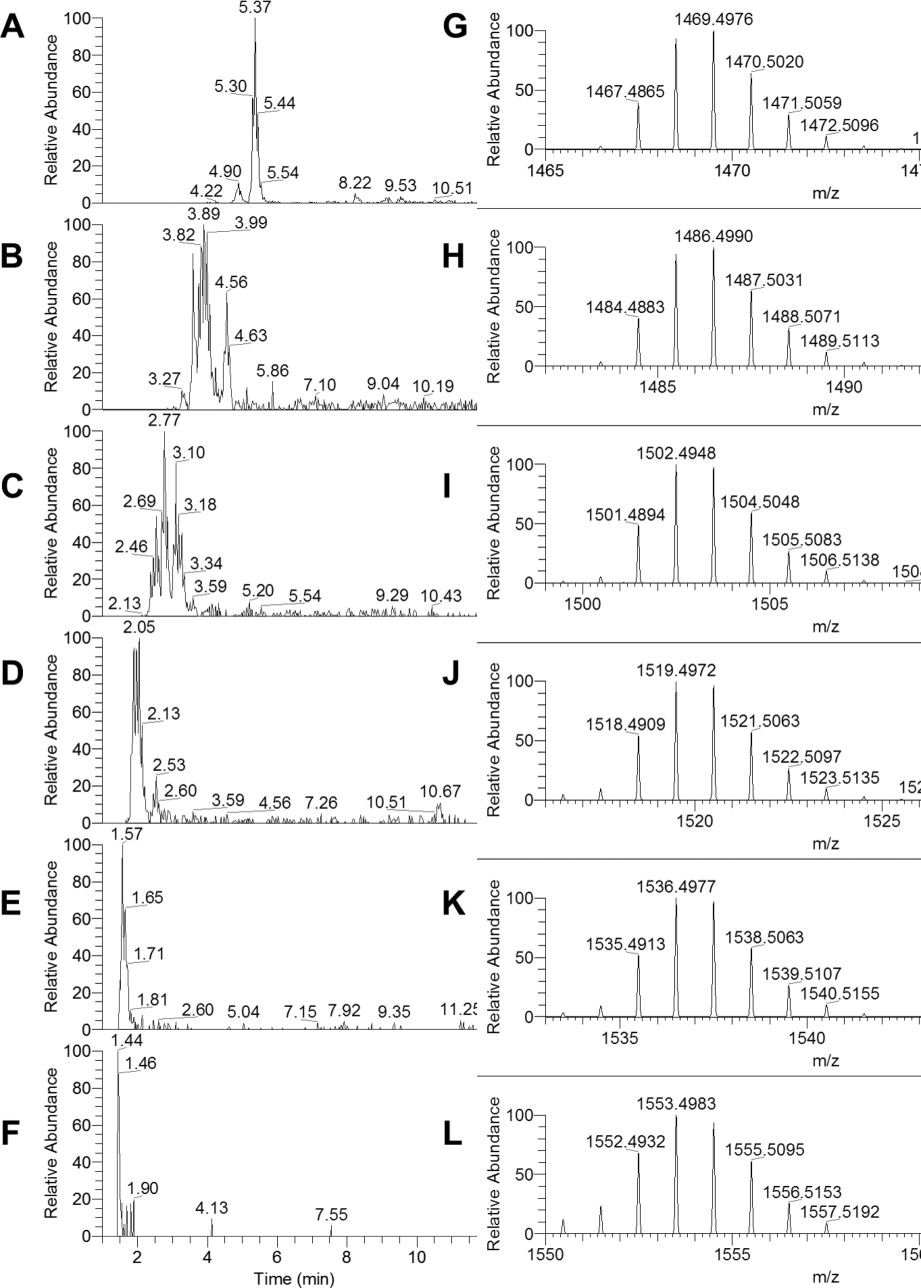
Extracted ion chromatograms in deuterium oxide for pentameric (A) *m*/*z* (D) 1467.4874 {0 (epi)gallocatechins, [C_75_H_35_D_25_O_30_ + D]^+^}, (B) *m*/*z* (D) 1484.4886 {1 (epi)gallocatechins, [C_75_H_34_D_26_O_31_ + D]^+^}, (C) *m*/*z* (D) 1501.4898 {2 (epi)gallocatechins, [C_75_H_33_D_27_O_32_ + D]^+^}, (D) *m*/*z* (D) 1518.4910 {3 (epi)gallocatechins, [C_75_H_32_D_28_O_33_ + D]^+^}, (E) *m*/*z* (D) 1535.4922 {4 (epi)gallocatechins, [C_75_H_31_D_29_O_34_ + D]^+^}, and (F) *m*/*z* (D) 1552.4934 {4 (epi)gallocatechins, [C_75_H_30_D_30_O_35_ + D]^+^}. Full MS for pentamers at (G) 5.4 min (for panel A), (H) 3.9 min (for panel B), (I) 2.8 min (for panel C), (J) 2.0 min (for panel D), (K) 1.6 min (for panel E), and (L) 1.4 (for panel F). A 4 ppm filter was applied to the extracted ion chromatograms.

To confirm the cyclic B-type structure of the identified species, HDX was applied to the wine sample prepared as described in the Experimental Section. In Figures 5 and 6, the HPLC–(HDX)–HRMS results for tetra- and pentamers are reported, respectively. With the HDX procedure, the species discussed in the previous paragraph were successfully assigned to cyclic (and non-cyclic) B-type proanthocyanidins, by comparison to the calculated theoretical masses associated.^1,4^ As in previous reports, a neat shift in retention times was observed for all species under HDX conditions. Indeed, D_2_O was successfully used for structural elucidations by exchange of weakly bound H atoms and was shown to delay the elution of analytes for most compounds because it owns different properties from H_2_O.^17^ In particular, D bonds in D_2_O are stronger than H bonds in H_2_O.^18^ Such a difference could probably be the reason for the delayed elution of such a PAC in D_2_O; however, the compound elution order was respected.

With regard to the results of the HDX, the number of exchanged protons was consistent with the theoretical B-type cyclic PACs (see Table 1). A direct comparison to the analogue and isobaric A-type oligomers for cyclic PC had also been performed for a peanut skin extract.^13^ A-type PACs with one A-type linkage, which are isobaric to the studied cyclic B-type PACs studied here, were predicted and successfully found to exchange one fewer proton to deuterium than the analogue cyclic B-type PACs and, therefore, unsuitable as possible assignment candidates although being isobaric. Moreover, non-cyclic A-type PACs showed usually similar or often delayed elution times than their direct B-type non-cyclic analogues,^13^ which is a very different behavior in comparison to the cyclic compounds investigated herein.

In conclusion, by applying HDX and MS/MS analysis, nine new macrocyclic crown B-type proanthocyanidins were partially identified. Four tetrameric prodelphinidins with 1–4 (epi)gallocatechins and five pentamers with 1–5 (epi)gallocatechins were observed. A consistent shift toward lower retention times was observed with the increasing proportion of (epi)gallocatechin monomeric units in the “crown” macrocyclic structure. Of these, the first (and most abundant) was the only fully isolated and characterized.^11^ Surely, the measured exact masses, the MS/MS spectra, the HDX shifts (consequential to the number of OH protons), and the relatively low number of isomers were all factors distinguishing these compounds from their non-cyclic B- or A-type analogues.^1^ As a future task, the full isolation and characterization of a number of these oligomers will be required, to identify similarities in the structures and conformation, which could, in turn, (1) shed light on the physicochemical properties of these compounds and their possible interactions with other components in wine, (2) provide hints of the mechanism(s) involved in their (bio)synthesis (and the condition favoring their formation versus the formation of non-cyclic analogues), (3) clarify their role in winemaking and in the assessment of the authenticity of wines, and (4) provide indications of their sensory properties.

## Supporting Information Available

Extracted ion chromatogram of *m*/*z* 1171.2714, full MS at 7.9 min, and MS/MS at 7.9 min of *m*/*z* 1171.271 precursor (Figure S1), extracted ion chromatogram of *m*/*z* 1187.2663, full MS at 7.0 min, and MS/MS at 7.0 min of *m*/*z* 1187.266 precursor (Figure S2), extracted ion chromatogram of *m*/*z* 1203.2612, full MS at 5.4 min, and MS/MS at 5.4 min of *m*/*z* 1203.261 precursor (Figure S3), extracted ion chromatogram of *m*/*z* 1459.3348, full MS at 8.5 min, and MS/MS at 8.5 min of *m*/*z* 1459.335 precursor (Figure S4), extracted ion chromatogram of *m*/*z* 1475.3297, full MS at 9.2 min, and MS/MS at 9.2 min of *m*/*z* 1475.330 precursor (Figure S5), and illustration of the QM and RDA fragmentation mechanisms (Figure S6) (PDF)
