# Supplementary material for: High-Performance Liquid Chromatography–Hydrogen/Deuterium Exchange–High-Resolution Mass Spectrometry Partial Identification of a Series of Tetra- and Pentameric Cyclic Procyanidins and Prodelphinidins in Wine Extracts
Source: J Agric Food Chem. 2020 Jan 13;68(11):3312–21. doi: 10.1021/acs.jafc.9b06195 (PMC7993638; doi:10.1021/acs.jafc.9b06195)
Supplement: Supplementary file 1 — jf9b06195_si_001.pdf [file jf9b06195_si_001.pdf]

SPE-enhanced HPLC-HDX-HRMS for the tentative identification of a series of tetrameric and pentameric cyclic procyanidins and prodelphinidins in wine

Merkytė, Vakarė,[1,2] Longo, Edoardo,[1,2] * Jourdes, Michaël,[3] Jouin, Alicia,[3] Teissedre, Pierre-Louis,[3] Boselli, Emanuele[1,2]

AFFILIATIONS

[1]Free University of Bozen-Bolzano, Faculty of Science and Technology, Piazza Università 5, 39100 Bozen-Bolzano, Italy

[2]Oenolab, NOI Techpark South Tyrol, Via A. Volta 13B, 39100 Bolzano, Italy

[3]Unité de Recherche Œnologie, EA 4577, USC 1366 INRA, ISVV, Université de Bordeaux, F33882 Villenave d'Ornon, France

*corresponding author: Dr Edoardo Longo (ORCID ID: 0000-0002-0594-6722)

Address: a) Free University of Bozen-Bolzano, Faculty of Science and Technology, piazza Università 5, 39100 Bolzano, Italy; Phone: +39 0471 017691, e-mail: edoardo.longo@unibz.it

SUPPORTING INFORMATION

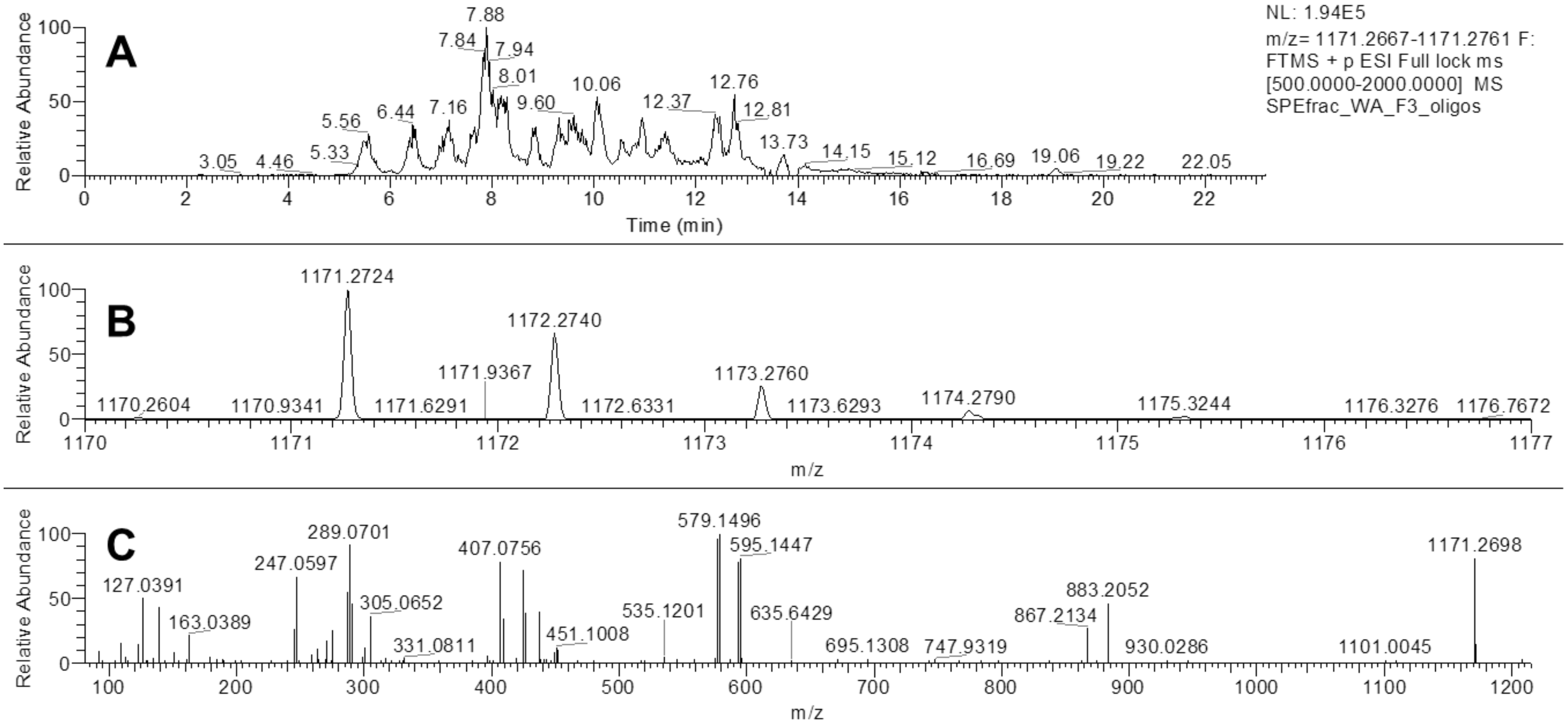

FIGURE SI 1 A) Extracted ion chromatogram of *m/z* 1171.2714 (non-cyclic tetramer with 1 (epi)gallocatechin, [$C_{60}H_{50}O_{25}$ + H]$^+$); B) Full MS at 7.9 min; C) MS/MS spectra at 7.9 min for *m/z* 1171.271. 4 ppm EIC filter applied. Spectra averaged over Δr.t. ≥ 1 min.

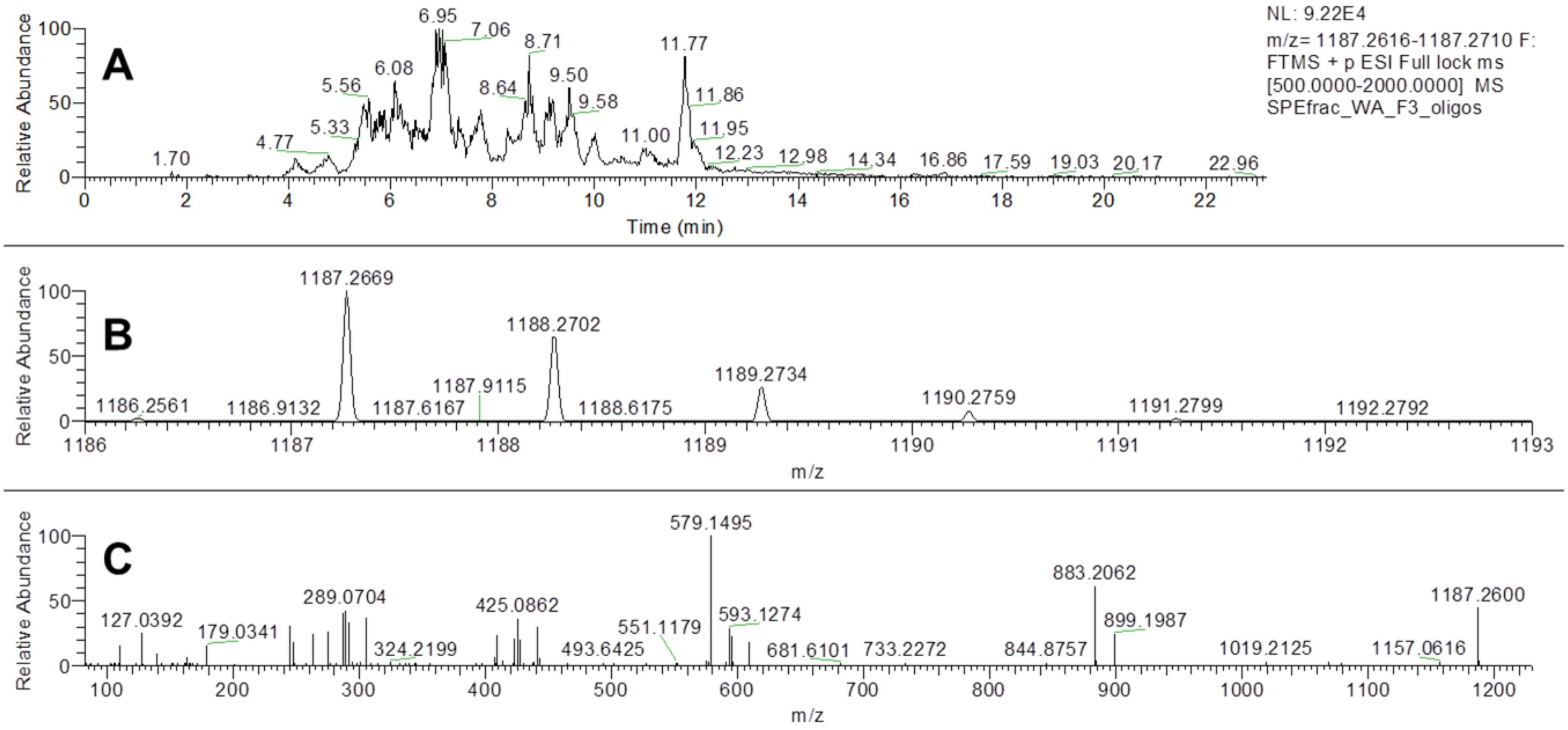

FIGURE SI 2 A) Extracted ion chromatogram of *m/z* 1187.2663 (non-cyclic tetramer with 2 (epi)gallocatechins, $[C_{60}H_{50}O_{26} + H]^+$); B) Full MS at 7.0 min; C) MS/MS spectra at 7.0 min for *m/z* 1187.266. 4 ppm EIC filter applied. Spectra averaged over Δr.t. ≥ 1 min.

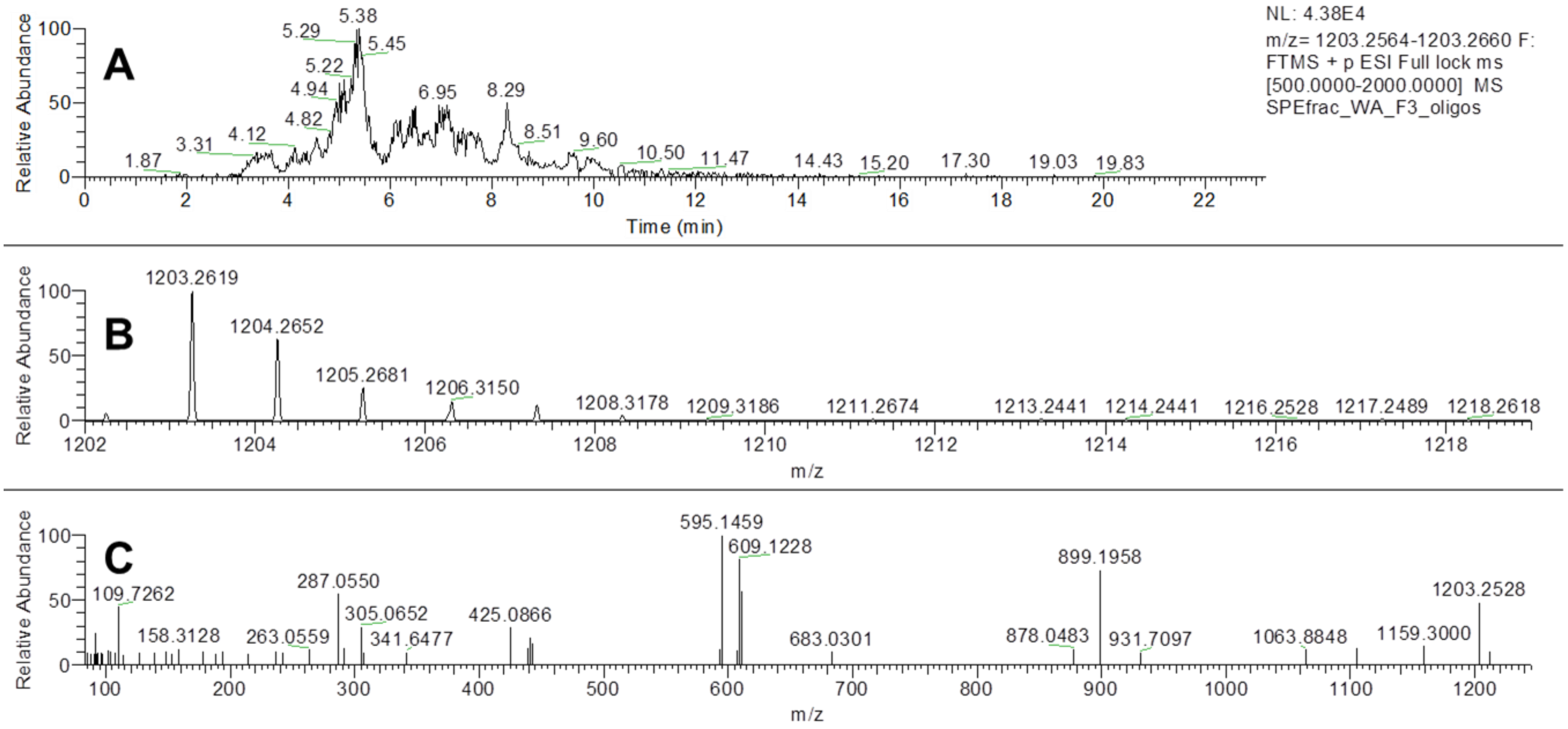

FIGURE SI 3 A) Extracted ion chromatogram of *m/z* 1203.2612 (non-cyclic tetramer with 3 (epi)gallocatechins, $[C_{60}H_{50}O_{27} + H]^+$); B) Full MS at 5.4 min; C) MS/MS spectra at 5.4 min for *m/z* 1203.261. 4 ppm EIC filter applied. Spectra averaged over Δr.t. ≥ 1 min.

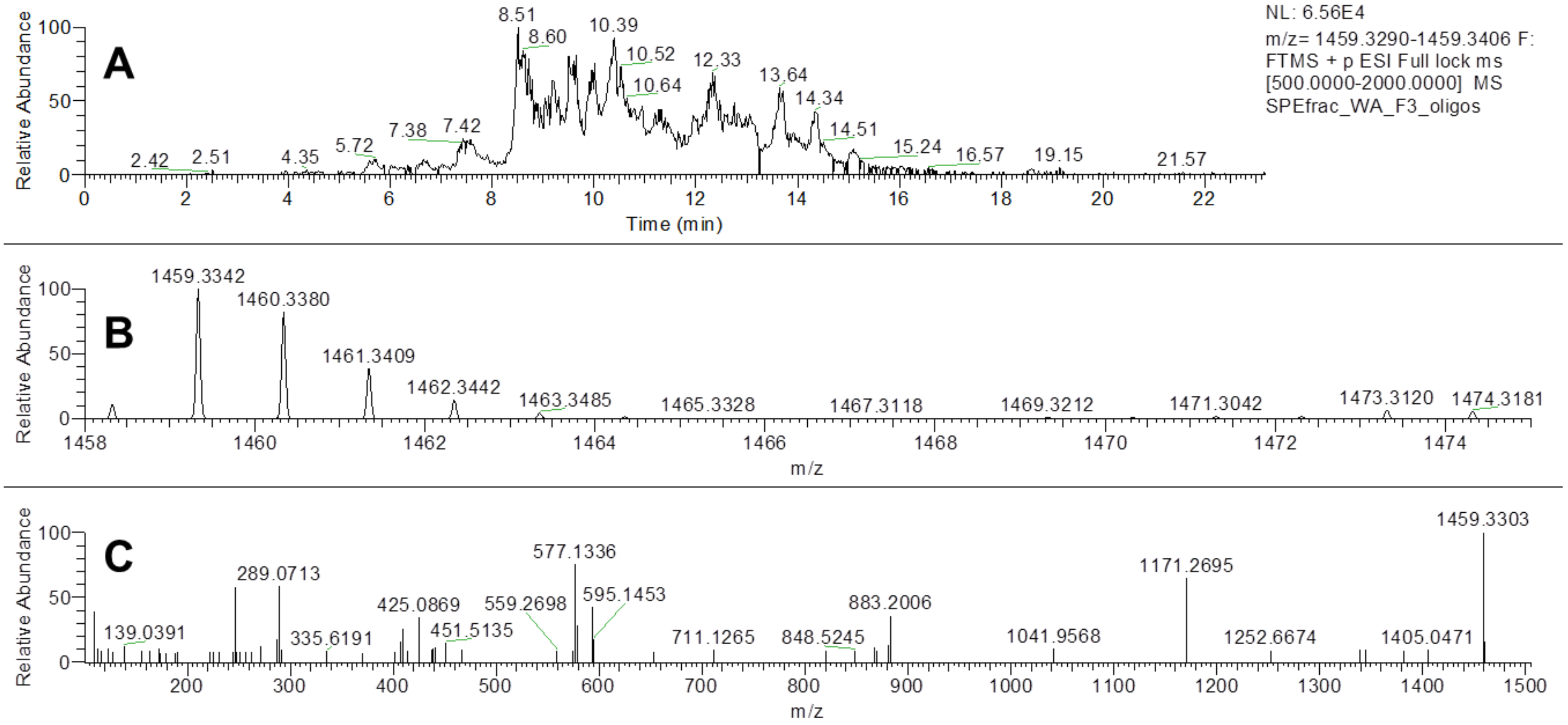

FIGURE SI 4 A) Extracted ion chromatogram of *m/z* 1459.3348 (non-cyclic pentamer with 1 (epi)gallocatechin, $[C_{75}H_{62}O_{31} + H]^+$); B) Full MS at 8.5 min; C) MS/MS spectra at 8.5 min for *m/z* 1459.335. 4 ppm EIC filter applied. Spectra averaged over Δr.t. ≥ 1 min.

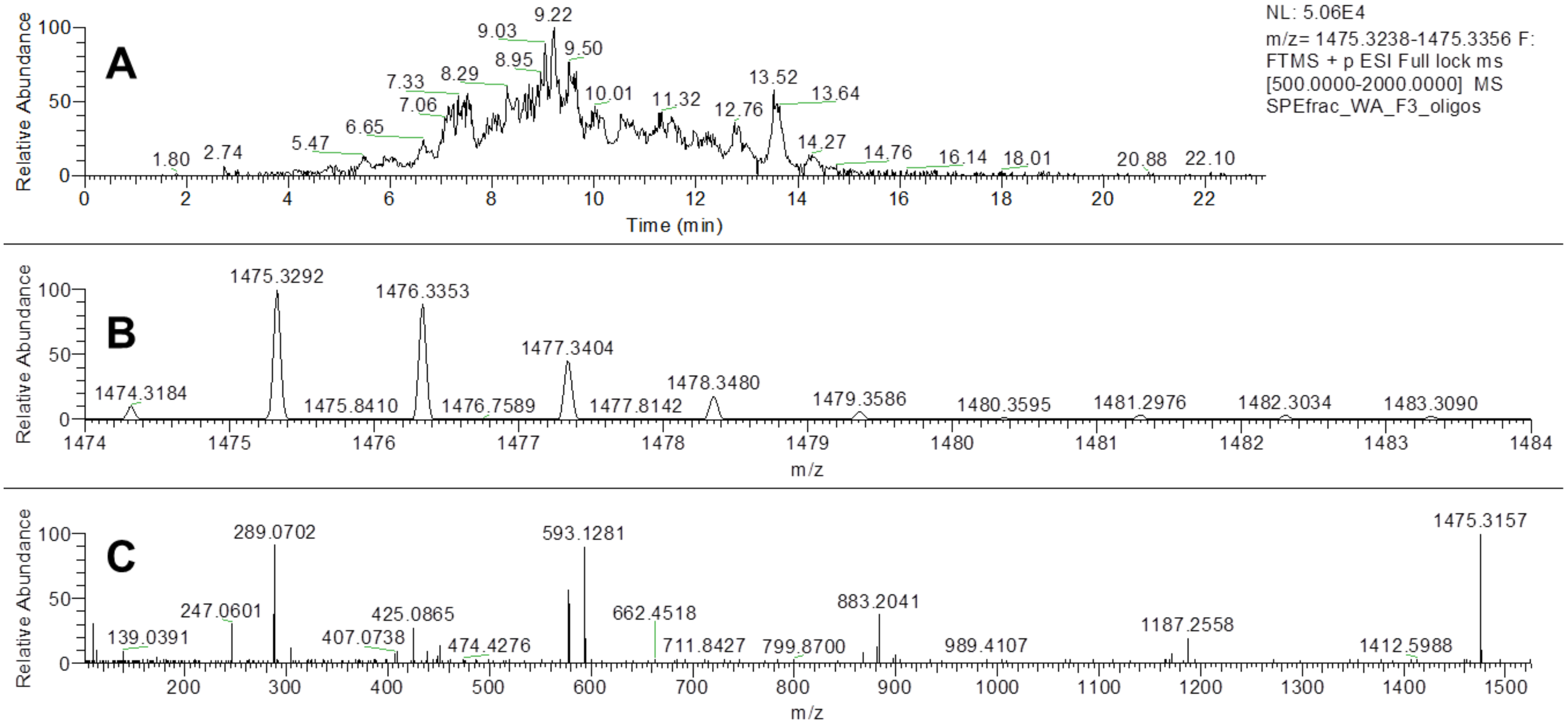

FIGURE SI 5 A) Extracted ion chromatogram of *m/z* 1475.3297 (non-cyclic pentamer with 2 (epi)gallocatechin, $[C_{75}H_{62}O_{32} + H]^+$); B) Full MS at 9.2 min; C) MS/MS spectra at 9.2 min for *m/z* 1475.330. 4 ppm EIC filter applied. Spectra averaged over Δr.t. $\geq$ 1 min.

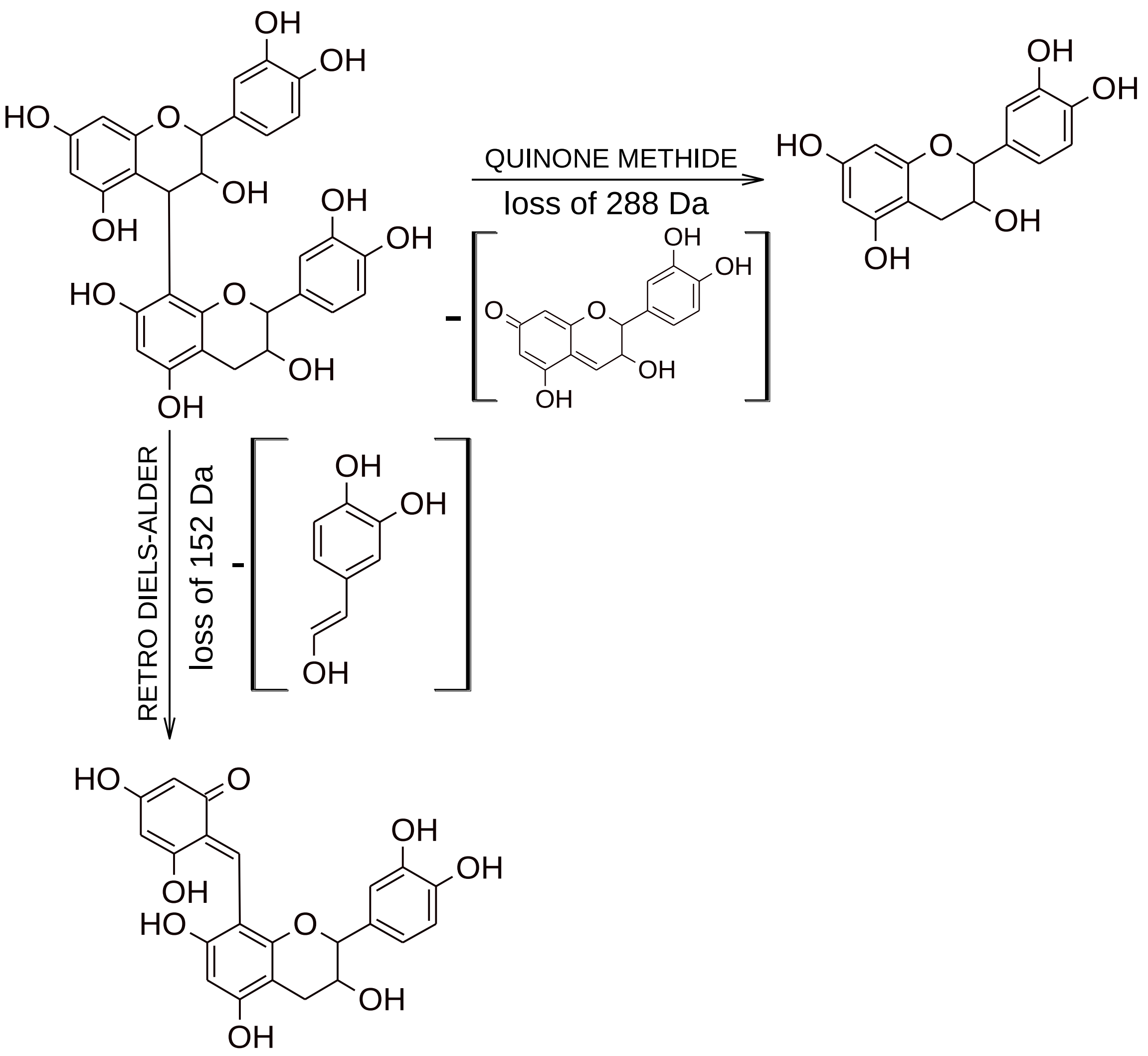

FIGURE SI 6 Examples of the MS/MS fragmentation patterns for c-PAC. In the Figure the main QM and RDA mechanisms are described for a model dimer procyanidin.
